# Supplementary material for: Pressure-induced reversal of Peierls-like distortions elicits the polyamorphic transition in GeTe and GeSe
Source: Nat Commun. 2023 Dec 7;14:7851. doi: 10.1038/s41467-023-43457-y (PMC10703813; doi:10.1038/s41467-023-43457-y)
Supplement: Supplementary file 1 — Supplementary Information [file 41467_2023_43457_MOESM1_ESM.pdf]

Supplementary Information

Pressure-induced reversal of Peierls-like distortions elicits the polyamorphic transition in GeTe and GeSe

Tomoki Fujita[1,10], Yuhan Chen[2,10], Yoshio Kono[3], Seiya Takahashi[4], Hidetaka Kasai[4], Davide Campi[5], Marco Bernasconi[5], Koji Ohara[6], Hirokatsu Yumoto[7,8], Takahisa Koyama[7,8], Hiroshi Yamazaki[7,8], Yasunori Senba[7,8], Haruhiko Ohashi[7,8], Ichiro Inoue[8], Yujiro Hayashi[8], Makina Yabashi[8], Eiji Nishibori[4], Riccardo Mazzarello[2], Shuai Wei[1,9]

[1]Department of Chemistry, Aarhus University, 8000 Aarhus C, Denmark.
[2]Department of Physics, Sapienza University of Rome, Rome 00185, Italy.
[3]Geodynamics Research Center, Ehime University, Matsuyama 790-8577, Japan.
[4]Department of Physics, Faculty of Pure and Applied Sciences and Tsukuba Research Center for Energy Materials Science (TREMS), University of Tsukuba, Ibaraki 305-8571, Japan.
[5]Department of Materials Science, University of Milano-Bicocca, I-20125 Milano, Italy.
[6]Faculty of Materials for Energy, Shimane University, Matsue, Shimane 690-8504, Japan.
[7]Japan Synchrotron Radiation Research Institute, 1-1-1 Kouto, Sayo-cho, Sayo-gun, Hyogo 679-5198, Japan.
[8]RIKEN SPring-8 Center, 1-1-1 Kouto, Sayo-cho, Sayo-gun, Hyogo 679-5148, Japan.
[9]iMAT Centre for Integrated Materials Research, Aarhus University, Denmark.
[10]These authors contributed equally: Tomoki Fujita, Yuhan Chen.
E-mail: riccardo.mazzarello@uniroma1.it (RM); shuai.wei@chem.au.dk (SW)

Supplementary Notes

Overview of data processing

The flowchart of the data processing is shown in Supplementary Fig. 1. First, we classified the measured diffraction data I(Q) into two groups based on the existence of Bragg peaks. The diffraction data without Bragg peaks were directly transferred for further data processing. For the partially crystallized data with Bragg peaks, we performed Rietveld refinement to split the I(Q) into the broad scattering predominantly from the amorphous state and the Bragg peaks from the crystalline state. Next, we applied the background subtraction, intensity correction, and normalization for the I(Q) of the amorphous state to convert it into the total structure factor S(Q). Finally, the reduced pair distribution function G(r) was obtained by the Fourier transformation of the S(Q).

Rietveld refinement

For the partially crystallized data of high-pressure GeTe, Rietveld refinement was performed to decompose the I(Q) into the crystalline contribution and the remaining broad scattering predominantly from the amorphous state. The data analysis was performed on the program Synchrotron Powder and Plotpro[1]. The analytical range was selected as 0.4° to 20° in 2θ. The split-type pseudo-Voigt function was used as the profile function for modelling the Bragg peaks. The remaining broad scattering was expressed as the sum of 9 profile functions. We employed the split-type PearsonVII functions for describing the asymmetric shape of the broad amorphous peaks. The profile parameters were optimized during the Rietveld refinement to achieve the best fit.

Supplementary Fig. 2 shows the representative results of Rietveld refinement. As shown in Supplementary Fig. 2a, Bragg peaks at high-pressure conditions are modelled by the rock-salt type cubic phase (space group $Fm\overline{3}m$) of GeTe. The difference between the observed and calculated intensity of Bragg peaks was only 0.2 % on average for the first 16 peaks (located at < 8°), which constitutes roughly 80 % of the total crystalline contribution. A slight deviation around 3.3° can be attributed to the scattering from a component of the sample cell. The agreement of overall analytical range was provided by the reliability factor R_{wp} = 4.18 % (lower values indicates the better reliability), suggesting that the remaining broad contribution was successfully expressed as the sum of 9 profile functions. Based on these results, we could successfully decompose the I(Q) into the crystalline contribution and the remaining broad scattering with sufficient accuracy. We note that the cubic phase was transformed into the stable rhombohedral phase (space group $R\overline{3}m$) at ambient pressure after pressure release, as clearly demonstrated by the peak split around 3.6° and the smaller separation between the 1st and 2nd peaks with respect to the cubic phase (Supplementary Fig. 2b)[2].

Total structure factor S(Q) and reduced pair distribution function G(r)

Proper corrections and normalization were applied to the I(Q) (raw data or the extracted broad contribution from Rietveld refinement) to obtain the total structure factor S(Q) and reduced pair distribution function G(r). All the procedures were performed on the software pdfgetX2[3]. The contribution from sample environment was subtracted from the scattering data. The profile of the sample environment was measured from an empty CDT cell (Supplementary Fig. 3a). In the present experimental setup, collimation slits placed in front of the sample can block the scattering from the sample environment such as the cell and surrounding air, achieving considerably low background intensity[4]. As shown in Supplementary Fig. 3a, the I(Q) of the empty cell suggested a small contribution from the sample environment, which was mainly composed of air scattering (less than 3 % above $Q >$ 2.0 Å^{-1} with respect to the intensity of sample scattering). The corrections for self-absorption, multiple scattering, and oblique incidence were applied to the I(Q) to convert it into S(Q). The energy-dependent Compton scattering (1/E quadratic form) was calculated. Supplementary Fig. 3a shows the I(Q) after

correction and the profile of Compton scattering. A Breit-Dirac factor of 2 was used for the Compton scattering. The parameters of Compton scattering were optimized to achieve $S(Q)\rightarrow 1$ for the Q-range from 20 to 25 Å^{-1}. Supplementary Fig. 3b shows the obtained total structure factor $S(Q)$, which was subsequently Fourier-transformed into the reduced pair distribution function $G(r)$. The Lorch function was used for reducing the truncation oscillations in $G(r)$. The peak positions of $S(Q)$ and $G(r)$, considered in the present study, are known to be robust with respect to the selection of the correction parameters[5].

The full width of half maximum (FWHM) of the first main diffraction peak of S(Q)

The evaluation of FWHM of the first main diffraction peak Q_1 in $S(Q)$ is interfered with the partial overlap with the neighboring second peak, which shifts towards the first peak with pressure increase. In the main text, FWHM is evaluated as the distance between two positions of Q at which $S(Q)$ becomes a half of $S(Q_1)$, possibly including some contribution from the second peak. To examine the robustness of the pressure dependence against the peak overlap, FWHM is estimated by fitting the first peak with a Lorentzian profile[6]. Supplementary Fig. 5a shows the representative results of fitting with two different Q-ranges: 1.5 Å^{-1} to Q_1 (denoted as low-Q) and 1.5 Å^{-1} to 2.5 Å^{-1} (high-Q). The fitting with low-Q range provided the lowest FWHM of 0.478 Å^{-1}, whereas half of $S(Q_1)$ resulted in the largest FWHM of 0.632 Å^{-1}. Supplementary Fig. 5b and 5c shows the pressure dependence of FWHM for GeSe and GeTe estimated by these three methods. Clearly, regardless of such a difference in absolute values of FWHM, the width exhibited essentially the same pressure dependence., i.e. a kink is observed around the transition pressure 3.5 GPa for GeSe, and FWHM of GeTe exhibited a distinct drop above 1.8 GPa. The results demonstrate that the coherent length is significantly increased after polyamorphic transition and the high-pressure state of GeSe and GeTe is characterized by the higher structure coherence.

The pressure environment of the Paris-Edinburgh press

The high-pressure X-ray scattering data in the present study were collected from the samples in the Paris-Edinburgh press. We used soft hexagonal BN and MgO as a pressure medium surrounding the sample, which is often used in large volume press experiments for quasi-hydrostatic environment. To investigate the effect of homogeneity of pressure environment, we compared the pressure dependence of the atomic volume ratio V/V_0 of the partially crystallized volume fraction of GeTe obtained in this study with the previous ones which were obtained under hydrostatic pressure conditions (Supplementary Fig. 6). V is the atomic volume calculated from the lattice parameters of cubic GeTe, and V_0 is the atomic volume at ambient pressure. The solid line is the third-order Birch-Murnaghan fit reported in the previous study under the hydrostatic pressure conditions by using the diamond anvil cell and ethanol-methanol-water mixture as a pressure medium[2]. In the present study, we observed the crystalline peaks of GeTe only above 3.4 GPa in the compression process. To compare the pressure response, the V_0 was estimated by using the volume ratio of the reported value at 3.4 GPa. All the data points are consistent up to 10.2 GPa with the reported curve within error bar, suggesting that the observed pressure response in the present pressure range should be close to the one that could have been observed under hydrostatic conditions.

Supplementary Figures

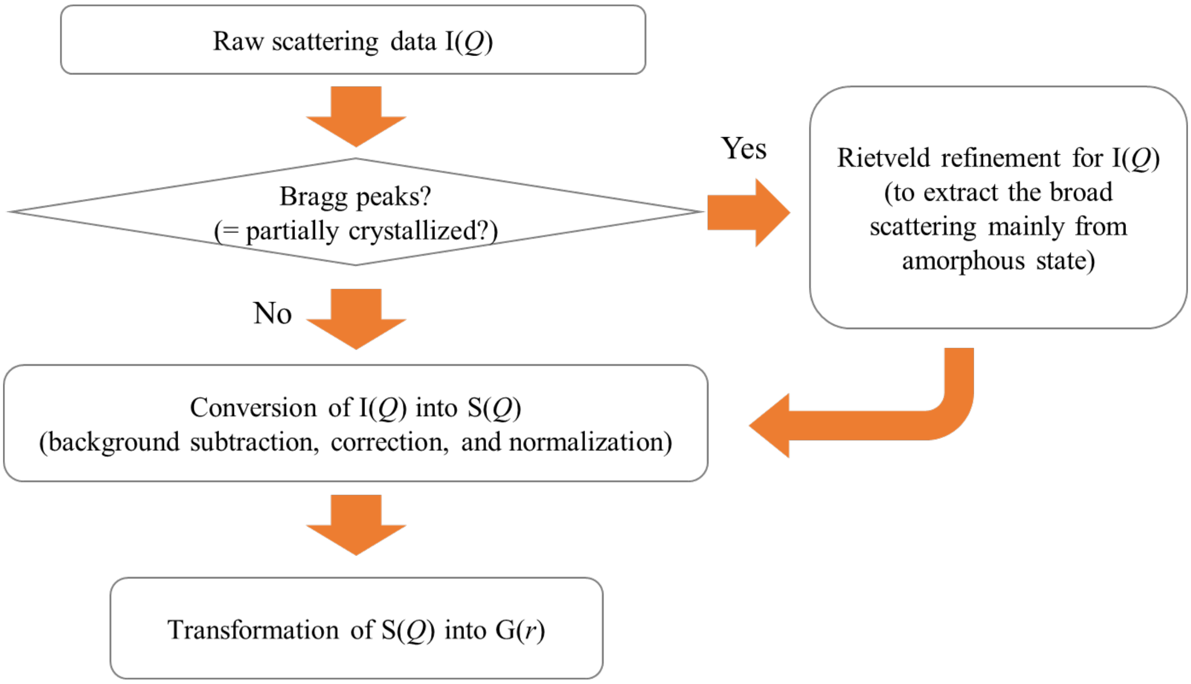

Supplementary Fig. 1 Flowchart of data processing to convert the raw diffraction data I(*Q*) into total structure factor S(*Q*) and reduced pair distribution function G(*r*).

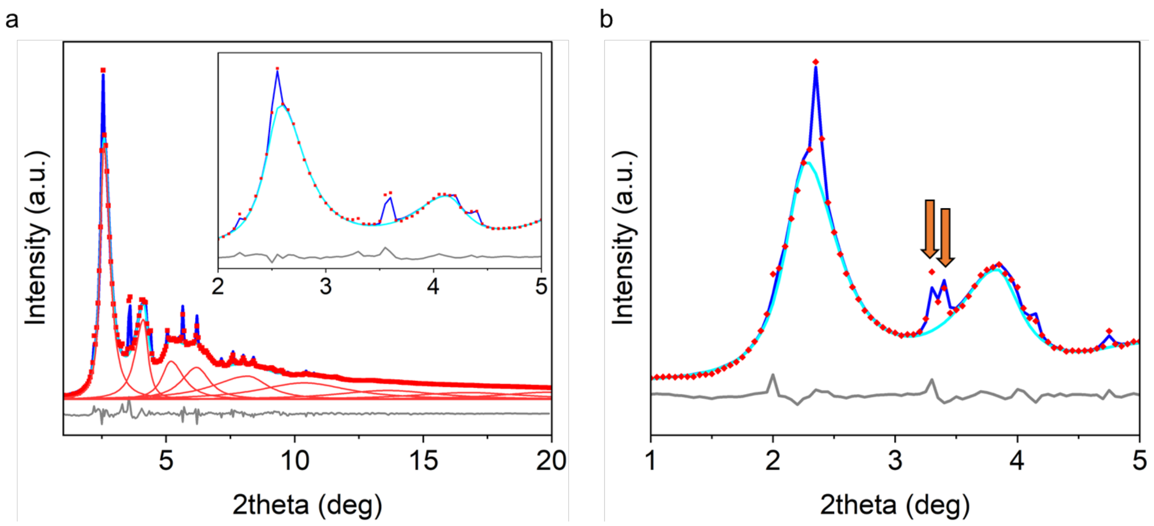

Supplementary Fig. 2 (a) The result of Rietveld refinement of GeTe at 10.2 GPa. The best fit was obtained by the cubic GeTe model with rock-salt structure. The experimental data (red dots), the calculated data (blue line), the contribution of 10 split-type Pearson-VII profiles (orange lines), and the residuals between experimental and calculated data (gray line). The inset is a magnification of diffraction angle from 2° to 5°. (b) The result of refinement for diffraction data after pressure release. Bragg peaks are modelled with the rhombohedral phase. The two orange arrows around 3.3° highlight (104) and $(2\bar{1}0)$ reflections that are iconic features of rhombohedral phase, in contrast with a single peak of cubic GeTe at 3.6° as shown in the inset of Supplementary Fig. 2a.

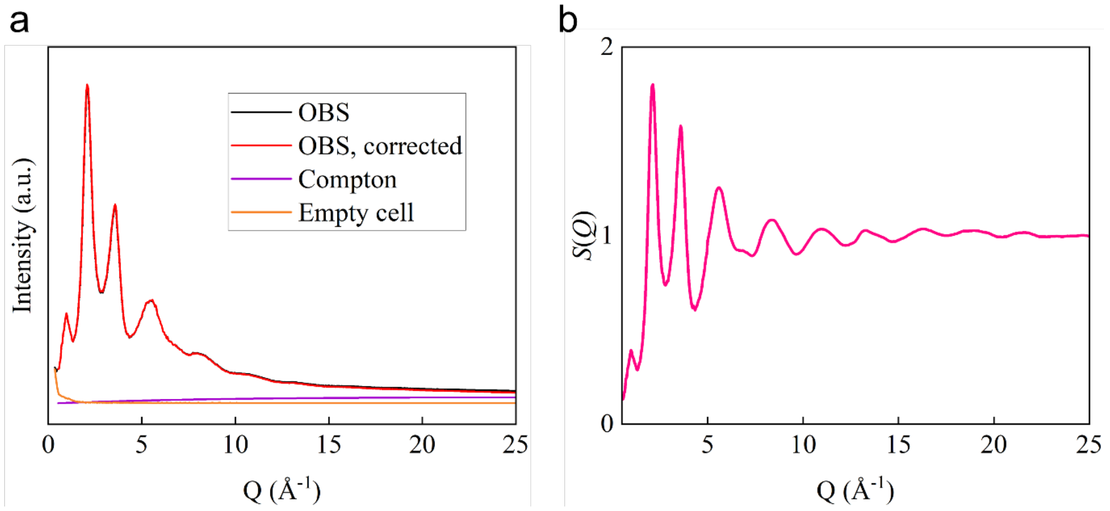

Supplementary Fig. 3 (a) I(Q) of amorphous GeSe at ambient pressure (black), the I(Q) after applying the correction for the self-absorption, multiple scattering, and oblique incidence(red), the profile of Compton scattering (purple) and the profile of empty cell (orange). (b) The total structure factor S(Q) of amorphous GeSe at ambient pressure.

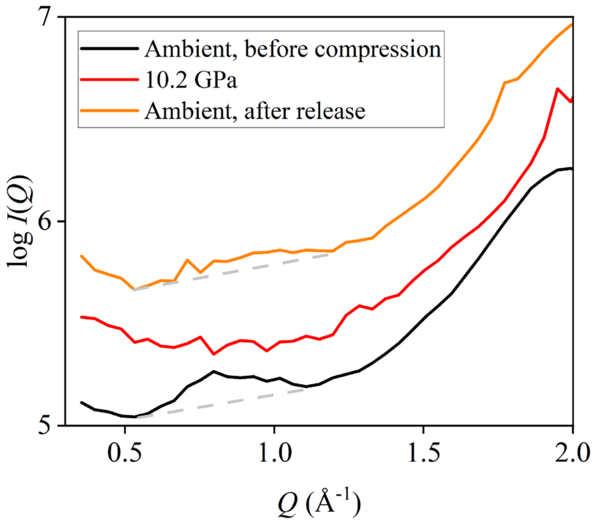

Supplementary Fig. 4 The diffraction intensity I(Q) of amorphous GeTe at ambient pressure before compression (black), at 10.2 GPa (red), and at ambient pressure after pressure release from 10.2 GPa (orange). The low-Q range was magnified to show the pre-peak. The existence of the pre-peak after pressure release indicates the recovery of Peierls-like distortion and indicates the reversibility of the polyamorphic transition.

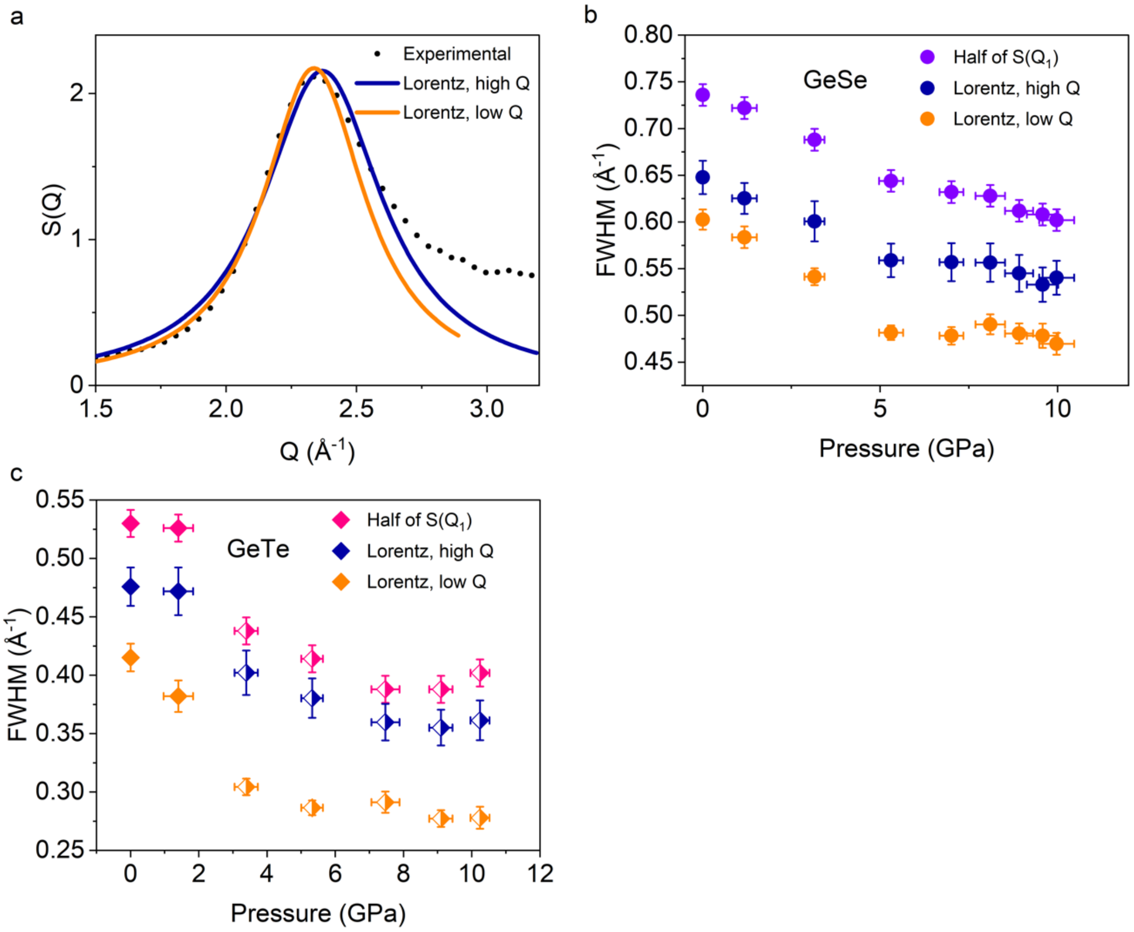

Supplementary Fig. 5 (a) The first main diffraction peak of total structure factor S(Q) of GeSe at 7.0 GPa, and the fitting curves by a Lorentzian profile. Two lines are the profiles by fitting two different Q-ranges: 1.5 $Å^{-1}$ to Q_1 (orange, denoted as low-Q) and 1.5 $Å^{-1}$ to 2.5 $Å^{-1}$ (navy, denoted as high-Q) (b)(c) The pressure dependence of FWHM of GeSe and GeTe.

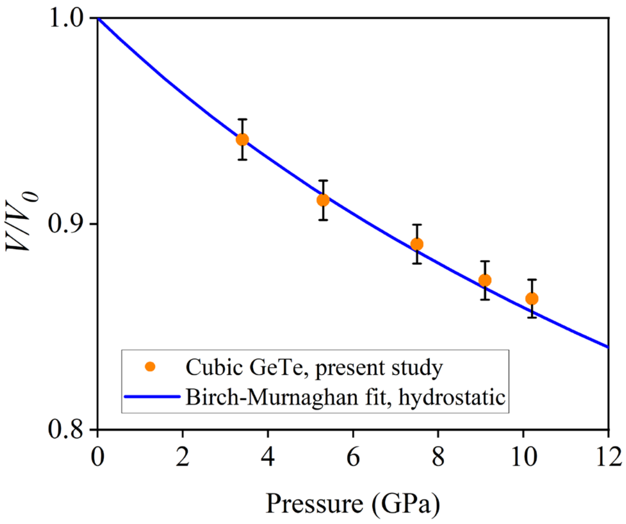

Supplementary Fig. 6 The pressure dependence of atomic volume ratio of the partially crystallized GeTe. The blue line is the third-order Birch-Murnaghan fit determined for the data points obtained under the hydrostatic pressure conditions[2]. The results of the present study are consistent with the reported line within the error.

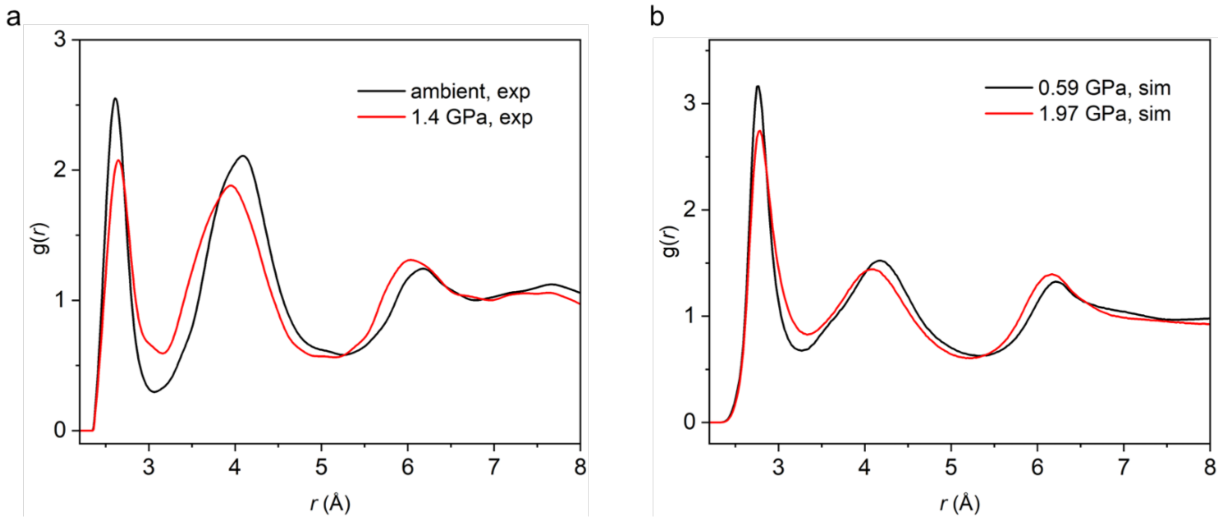

Supplementary Fig. 7 Pressure dependence of the pair correlation functions g(*r*) of GeTe obtained from (a) experiment and (b) MD simulation. The simulations qualitatively reproduce the pressure dependence of g(*r*) obtained from the experiments.

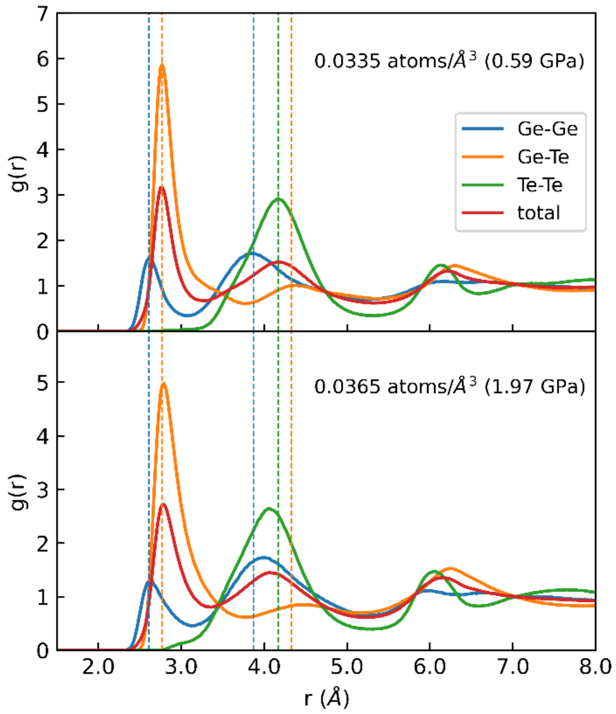

Supplementary Fig. 8 Partial pair correlation functions of amorphous GeTe at pressure 0.59 GPa (density 0.0335 atoms/ Å^3) and 1.97 GPa (0.0365 atoms/ Å^3). The first peak is due to Ge-Te bonds and, to a lesser extent, Ge-Ge bonds, whereas the second peak is mainly due to Te-Te contacts. All g(r) curves are normalized such that g(r) approaches 1.0 at large *r* values.

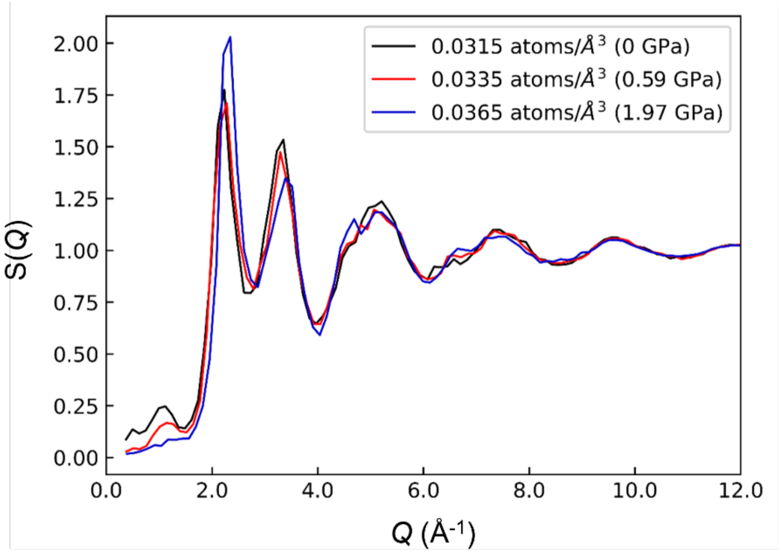

Supplementary Fig. 9 S(Q) of amorphous GeTe at zero pressure and at pressure 0.59 GPa and 1.97 GPa. The low-pressure curves exhibit a pre-peak, whereas the pre-peak disappears at 1.97 GPa due to the suppression of Peierls-like distortion.

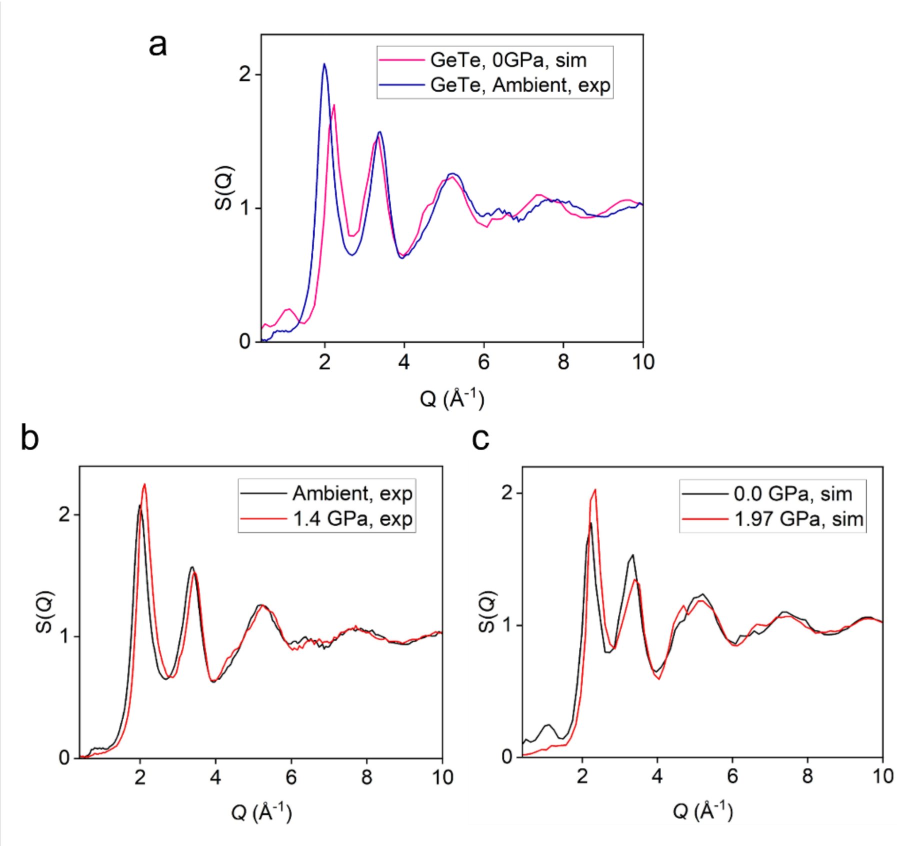

Supplementary Fig. 10 a) A comparison between the experimental and computational S(Q) of GeTe without pressure. There are some quantitative discrepancies: in particular, the computational first peak is lower than the experimental one and is shifted to higher Q. Nevertheless, such discrepancies are in line with those found in the literature between experimental S(Q) and those obtained from ab initio melt-quenched models of PCMs based on standard DFT functionals. Qualitatively, the simulations reproduce the main features of the experimental S(Q), including the presence of the pre-peak. (b-c) The pressure dependence of the total structure factor S(Q) of GeTe obtained from experiment and MD

simulation, respectively. The simulations qualitatively reproduce the pressure dependence of S(Q) obtained from the experiments, including the vanishing of the pre-peak around 1.0 Å^{-1}.

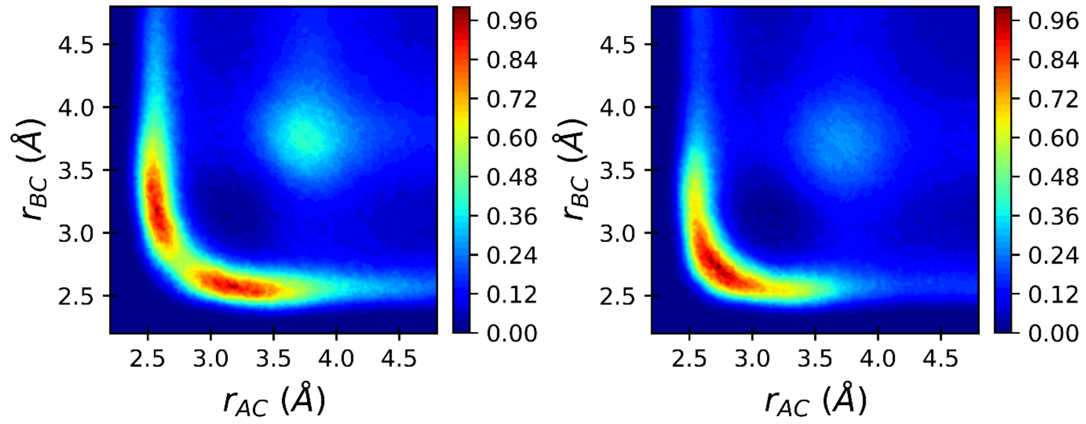

Supplementary Fig. 11 Angular limited three body correlation (ALTBC) plots of GeSe at 2.42 GPa (left) and 5.17 GPa (right) showing suppression of Peierls-like distortion for increasing pressure. Comparison with the GeTe data (Figure 5 in the main text) indicates that the polyamorphic transition occurs at higher pressure as compared to GeTe, in agreement with experimental data.

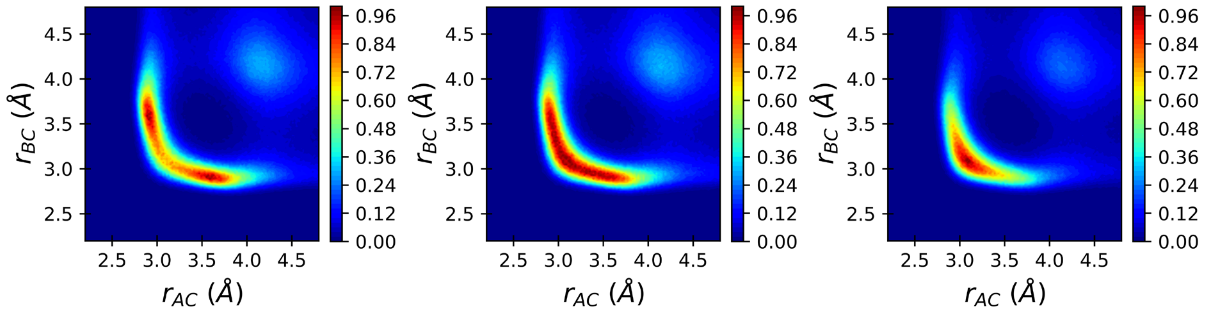

Supplementary Fig. 12 The ALTBC distribution of amorphous antimony at the pressure of 0.22, 0.37 and 0.55 GPa.

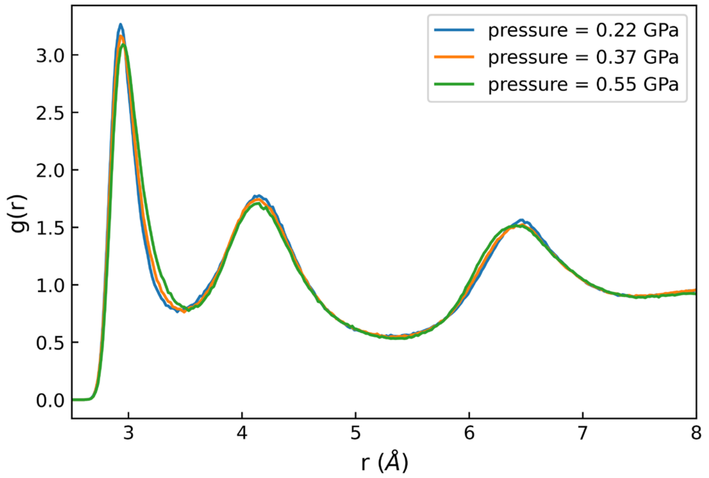

Supplementary Fig. 13 The pair correlation functions g(*r*) of amorphous antimony at different pressures. By increasing the pressure (density), the first two peaks of g(*r*) tend to approach each other, indicating the decrease of *R*. Precisely, the value of *R* changes from 1.42 (0.22 GPa) to 1.40 (0.55 GPa). It is not feasible to consider higher pressures due to the fast crystallization.

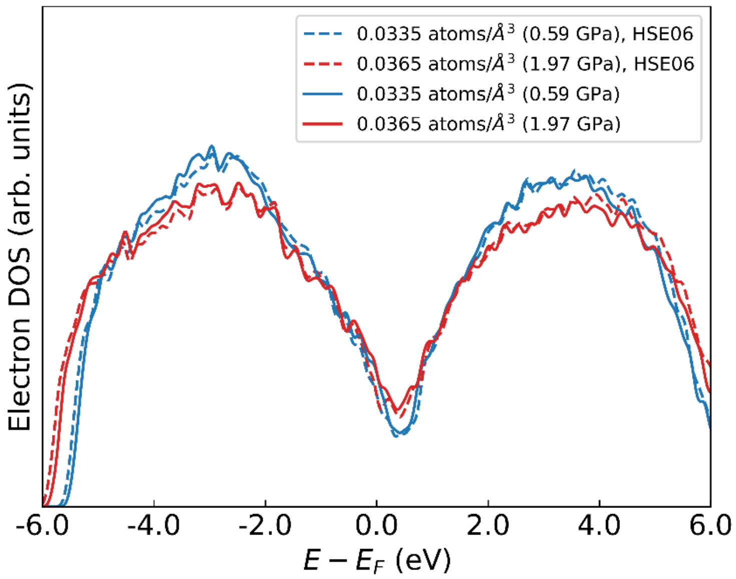

Supplementary Fig. 14 Comparison between the density of states of GeTe at two different pressures calculated using the TASK functional (continuous lines) and the hybrid HSE06[8] functional (dashed lines). The results hardly depend on the functional. The same basis set and cutoffs were used in the two sets of calculations. The hybrid-functional calculations were accelerated by using the auxiliary density matrix method (ADMM)[9].

Supplementary Tables

Pressure (GPa)	0	0.59	0.97	1.97
Fraction of $Ge^{Tetra.}$ motifs	36.3%	33.3%	29.2	21.5%

Supplementary Table 1 The fraction of Ge atoms with tetrahedral coordination ($Ge^{Tetra.}$) in amorphous GeTe as a function of pressure. A tetrahedral structure corresponds to a value of the bond order parameter q between 0.8 and 1.0. The bond order parameter[7] of an atom k is defined as $q = q_k = 1 - \frac{3}{8}\sum_{i=1}^{N_k^{nb}} \sum_{j=i+1}^{N_k^{nb}} \left(\frac{1}{3} + \cos\theta_{ikj}\right)^2$, where θ_{ikj} is the angle between the vectors connecting the atom k to atoms i and j. N_k^{nb} denotes the number of nearest neighbors of the atom k, which is determined by the cutoffs for each specific pair: 3.0, 3.22 and 3.0 Å for Ge-Ge, Ge-Te and Te-Te respectively.

Supplementary References

1. Nishibori, E. *et al.* Accurate structure factors and experimental charge densities from synchrotron X-ray powder diffraction data at SPring-8. *Acta Crystallogr. A* **63**, 43–52 (2007).
2. Onodera, A., Sakamoto, I., Fujii, Y., Mo*ₖ*ri, N. & Sugai, S. Structural and electrical properties of GeSe and GeTe at high pressure. *Phys. Rev. B* **56**, 7935–7941 (1997).
3. Qiu, X., Thompson, J. W. & Billinge, S. J. L. PDFgetX2: a GUI-driven program to obtain the pair distribution function from X-ray powder diffraction data. *J. Appl. Crystallogr.* **37**, 678–678 (2004).
4. Kono, Y. *et al.* Experimental evidence of tetrahedral symmetry breaking in SiO_2 glass under pressure. *Nat. Commun.* **13**, 2292 (2022).
5. Gallington, L. C., Wilke, S. K., Kohara, S. & Benmore, C. J. Review of Current Software for Analyzing Total X-ray Scattering Data from Liquids. *Quantum Beam Sci.* **7**, 20 (2023).
6. Ryu, C. W. & Egami, T. Origin of liquid fragility. *Phys. Rev. E* **102**, 042615 (2020).
7. Errington, J. R., Debenedetti, P. G., Relationship between structural order and the anomalies of liquid water. Nature **409**, 318–321 (2001).
8. Heyd, J., Scuseria, G. E. & Ernzerhof, M., Hybrid functionals based on a screened Coulomb potential. *J. Chem. Phys.* **118**, 8207–8215 (2003).

9. Guidon, M., Hutter, J., VandeVondele, J., Auxiliary Density Matrix Methods for Hartree-Fock Exchange Calculations. *J. Chem. Theory Comput.* **6**, 2348–2364 (2010).
